# Nonlinear association of body roundness index with female infertility and the mediating effect of NHHR: A cross-sectional study

**DOI:** 10.1097/MD.0000000000046768

**Published:** 2025-12-19

**Authors:** WeiJing Yang, YaLu Fu, XingLong Liu, YuChan Wang, XiangYan Li, ZhanHong Du, YuHan Meng

**Affiliations:** a Department of Obstetrics and Gynecology, Puding County People’s Hospital, Anshun, Guizhou, People’s Republic of China; b Department of Reproductive Medicine, Affiliated Hospital of Shandong Second Medical University, Weifang, Shandong, People’s Republic of China; c School of Clinical Medicine, Shandong Second Medical University, Weifang, Shandong, People’s Republic of China; d Department of Orthopedics, Affiliated Hospital of Shandong Second Medical University, Weifang, Shandong, People’s Republic of China.

**Keywords:** BRI, cross-sectional analysis, infertility, NHANES, NHHR, prediction models

## Abstract

The Body Roundness Index (BRI) is an innovative anthropometric measure reflecting body shape and obesity, increasingly recognized as a potential predictor of infertility, warranting further exploration. This research employed a cross-sectional approach using National Health and Nutrition Examination Survey data collected between 2013 and 2018. A weighted multivariate regression model was applied to examine the connection between BRI and infertility risk. To analyze nonlinear trends, weighted restricted cubic splines were implemented, while generalized additive models helped detect threshold effects and inflection points. Subgroup and interaction analyses, adjusted by weights, evaluated specific subgroup roles. Mediation analysis investigated how non-HDL to HDL cholesterol ratio (NHHR) (non-HDL/HDL cholesterol ratio) acts as a mediator. The dataset was split into 70% training and 30% testing subsets. Characteristic variables were identified using LASSO regression, logistic regression, and the BORUTA algorithm. Prediction models were validated in both training and testing datasets to assess their predictive performance. The study analyzed data from 2576 participants. Infertility risk rose by 8% per unit increase in BRI after adjusting for covariates (odds ratio [OR] = 1.08; 95% confidence interval [CI]: 1.02–1.16; *P* = .018). Categorizing BRI into tertiles showed higher infertility risks for the second (OR = 1.71; 95% CI: 1.03–2.83; *P* = .038) and third tertiles (OR = 2.46; 95% CI: 1.37–4.43; *P* = .005) compared to the first tertile. Weighted restricted cubic spline analysis indicated a nonlinear link between BRI and infertility, while threshold effect analysis revealing a significant correlation for BRI < 6.4664 (*P* < .001). Weighted subgroup analysis showed significant interactions between BMI, age, and BRI. Mediation analysis demonstrated that NHHR mediates the association between BRI and infertility. Prediction models incorporating selected characteristic variables showed good clinical utility in both the training and testing datasets. BRI positively correlates with infertility risk, mediated by NHHR. Machine learning models suggest that incorporating BRI with other predictors can improve the management of high-risk infertility populations.

## 1. Introduction

Infertility denotes the incapacity to get pregnant after a year of regular, unprotected copulation.^[1,2]^Infertility has become a common international health problem, whether in industrialized and developing nations.^[3]^Currently, approximately 15% of couples worldwide are troubled by infertility, and the proportion is increasing year by year.^[4,5]^Infertility not only leads to the failure of couples’ reproductive wishes, but also has a negative impact on family relationships. It causes tension, anxiety and quarrels between couples, and even leads to marital crises. At the same time, the cost of related treatments also brings huge economic pressure to families. In addition, infertility also enhances the probability of diseases such as generative system tumors and metabolism-related illness.^[6–9]^It is well known that infertility is a multi-factor related disease, including environmental pollution, unhealthy lifestyles, obesity, and so on.^[10–12]^Among them, the risk factor obesity is recognized to be connected to the various diseases, including diabetes and cardiovascular conditions. Currently, more and more obese women are suffering from infertility.^[13,14]^ At present, obesity is commonly defined as a body mass index (BMI) ≥ 30 kg/m^2,[15]^ but this definition method has certain limitations. Existing studies have shown a significant association between BMI and female infertility, with the risk of infertility notably increasing in overweight and obese conditions.^[15]^ Although BMI can provide a general estimate of body weight, it does not assess the ratio of fat to muscle or the distribution of fat and muscle across the body. Consequently, BMI alone cannot accurately reflect an individual’s body fat level or overall health status. Reliance solely on BMI for obesity assessment is insufficient and lacks comprehensiveness; therefore, multiple indicators should be considered for a more accurate evaluation.^[3,16,17]^ The Body Roundness Index (BRI) is a new obesity metric that has been shown to offer greater accuracy compared to the traditional BMI. The BRI formula incorporates height and waist circumference, allowing a precise analysis of body fat and visceral fat ratio.^[18]^ While some research teams have begun exploring the relationship between BRI and infertility, further investigation into this area is warranted.

Cholesterol serves not only as a precursor for certain steroid hormones but also reflects changes in serum lipids associated with obesity. The non-HDL to HDL cholesterol ratio (NHHR) is a new lipid marker, significantly associated with cardiovascular disease risk.^[19]^ This study explores whether NHHR mediates the link between BRI and infertility.

## 2. Methods

### 2.1. Study population

**Figure 1.**
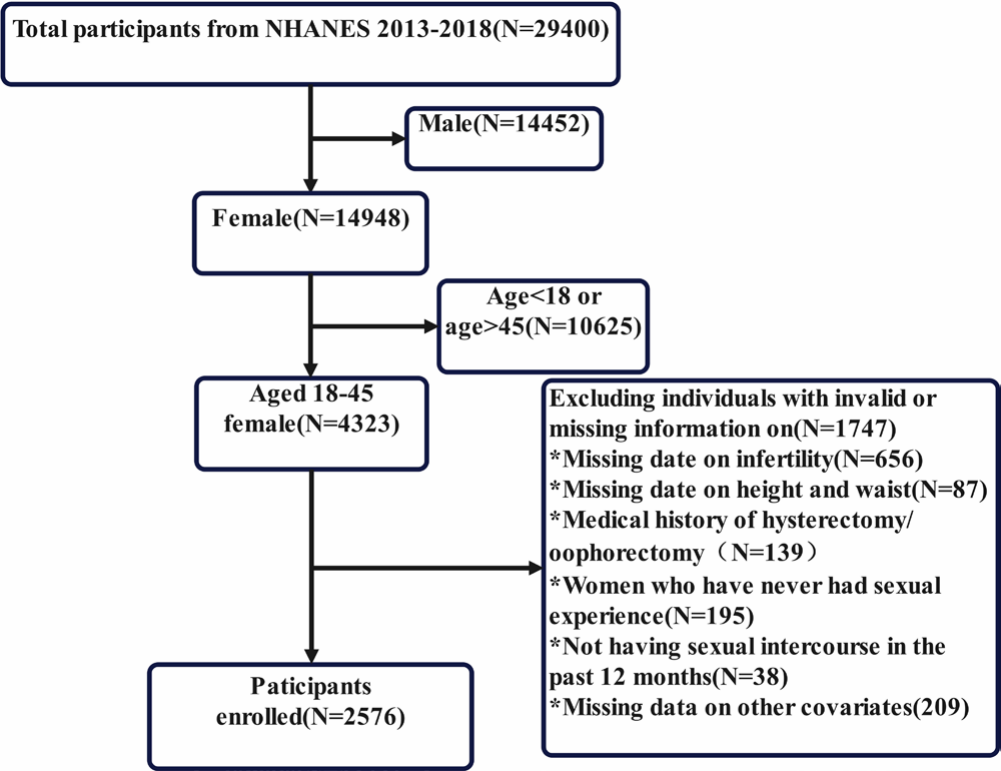
Flow chart for participants selection.

The study population was sourced from National Health and Nutrition Examination Survey (NHANES), a nationally representative survey using a complex multistage sampling design in the United States.^[20]^ All participants gave informed consent, and the survey adhered to STROBE guidelines.^[21]^ From the 29,400 participants enrolled between 2013 and 2018, the following exclusion criteria were applied: Male participants (N = 14,948), age below 18 years or above 45 years (N = 10,625), missing data on infertility status (N = 656), missing data on height or waist circumference (N = 87), and missing covariate data, lack of sexual activity, or absence of sexual activity within the past 12 months, hysterectomy, or bilateral oophorectomy (N = 508). After applying these exclusions, the final study cohort included 2576 participants, with the selection process detailed in Figure 1. The Ethics Review Board of the National Center for Health Statistics (NCHS) authorized all NHANES protocols, and written informed consent was obtained from all participants prior to their involvement. Furthermore, NCHS granted permission for the public release of the data.

### 2.2. Calculation of BRI

Height and waist circumference data were measured by trained staff at mobile examination centers. After converting the units to meters, BRI was computed using the formula: BRI = 364.2–365.5 × (1-[WC(m)/2π]^2^/[0.5 × Height(m)]^2^)^½^.^[22]^

### 2.3. Definition of infertility

The infertility factor that contributed was derived from the questionnaire dataset RHQ074 in the NHANES database. The main question was “Have you ever spent a year or more trying to conceive but failed to do so?” If the answer was “Yes,” it indicated the presence of infertility. If the answer was “No,” it indicated no infertility.^[23]^

### 2.4. Relevant covariates

Based on previous studies and clinical expertise, the following covariates were included in this study: age, race, education level, marital status, poverty-to-income ratio (PIR). History of hypertension, diabetes, pelvic inflammatory disease, and sleeping troubles. Contraceptive drug use, age at menarche, and menstrual regularity. Total cholesterol (TC) and high-density lipoprotein cholesterol (HDL-C). Physical activity level, sedentary behavior (defined as sitting for more than 6 hours per day), Drinking status (≥12 drinks/year), and smoking status (never smokers: <100 cigarettes lifetime; past smokers: ≥100 cigarettes but not current; current smokers: daily or occasional smoking).^[24]^

### 2.5. Statistical analysis

During the research process, the suggestions of the Centers for Disease Control and Prevention were adopted.^[25]^ Some of the analyses incorporated the sampling weights of NHANES for analysis to make the results more accurately reflect the specific situation of the Americans’ population. The population was divided into 2 sections according to the RHQ074 results of the participants. In the data analysis for the description of baseline characteristics, the expression for categorical variables was “sample size (weighted %),” with P values from survey-weighted Chi-square tests. Continuous variables were expressed as “Mean ± SD,” with P values from survey-weighted t-tests. Weighted multivariate logistic regression with 3 models analyzed the link between BRI and infertility risk. The crude model included no covariates, Model 1 adjusted for age and ethnicity. While Model 2 further incorporated extra covariates, including education, marital, PIR, diabetes, hypertension, smoking situation, drinking status, physical activity, sleep troubles, sedentary behavior, age at menarche, menstrual regularity, contraceptive drug use, TC, HDL-C, and history of pelvic inflammatory disease. Trend tests ensured result robustness. Nonlinear relationships between BRI and infertility risk were explored using weighted restricted cubic spline analysis, and threshold effects were assessed through weighted generalized additive model regression. Weighted subgroup analyses further evaluated potential interactions across various strata. Mediation analysis examined NHHR’s role in the BRI-infertility link.

To address class imbalance, the dataset was resampled using the synthetic minority oversampling technique, and the dataset was individed into training and testing sets in a 7:3 ratio. LASSO regression was used on the training set to identify potential features. Variables with non-0 regression coefficients under the optimal lambda penalty were selected as preliminary candidates.^[26,27]^ These variables were further refined through multivariate logistic regression and the Boruta algorithm. Cross-validation techniques and clinical insights were employed to finalize the feature selection. Key features (age, BRI, marital status, PIR, NHHR, HDL-C) were used to build a predictive model with the CatBoost algorithm. Model performance was assessed using ROC curves, calibration plots, and decision curve analysis to evaluate discrimination, calibration, and clinical utility. Statistical analyses were conducted in R (http://www.R-project.org) and EmpowerStats (www.empowerstats.com), with significance set at *P* < .05.

## 3. Results

### 3.1. Weighted baseline characteristics of the study population

Table 1 summarizes the baseline characteristics of the study cohort. Table S1, Supplemental Digital Content, https://links.lww.com/MD/R17 shows the full results of the weighted analysis, while Table S2, Supplemental Digital Content, https://links.lww.com/MD/R17 provides data from the unweighted analysis. Of the 2576 participants, 2275 were in the fertility group and 301 in the infertility group. Participants in the infertility group were significantly older, with a mean age of 35 years compared to 31.11 years in the fertility group (*P* < .001). The mean BRI was notably higher in the infertility group (6.35) compared to the fertility group (5.26) (*P* < .001). The NHHR was elevated in the infertility group, with a mean of 2.54 compared to 2.30 in the fertility group (*P* = .001).

**Table 1:** Characteristics of selected participants from the NHANES 2013–2018.

Characteristic	Overall N = 2576	Fertile N = 2275	Infertile N = 301	*P*-value
Age (yr)	31.59 ± 7.71	31.11 ± 7.70	35.00 ± 6.94	<.001
18 to <30	1090 (43.56%)	1013 (46.37%)	77 (23.59%)	
30 to ≤ 35	472 (18.01%)	415 (17.66%)	57 (20.56%)	
>35	1014 (38.43%)	847 (35.98%)	167 (55.85%)	
Race (%)				.600
Mexican American	431 (11.39%)	384 (11.58%)	47 (10.06%)	
Other Hispanic	266 (7.55%)	242 (7.75%)	24 (6.12%)	
Non-Hispanic White	891 (57.65%)	775 (57.02%)	116 (62.15%)	
Non-Hispanic Black	542 (12.71%)	478 (12.76%)	64 (12.40%)	
Non-Hispanic Asian	304 (5.77%)	269 (5.91%)	35 (4.78%)	
Other race	142 (4.92%)	127 (4.98%)	15 (4.49%)	
Education levels (%)				.92
Less than 9th grade	401 (11.31%)	360 (11.39%)	41 (10.68%)	
High school or equivalent	552 (20.14%)	494 (20.17%)	58 (19.93%)	
College or over	1623 (68.55%)	1421 (68.43%)	202 (69.39%)	
PIR Physical activity	2.69 ± 1.65	2.66 ± 1.65	2.89 ± 1.66	.05
				.340
Light activities	1135 (38.73%)	990 (38.09%)	132 (43.33%)	
Moderate activities	1195 (51.48%)	1063 (51.84%)	145 (48.85%)	
Vigorous activities	246 (9.79%)	222 (10.07%)	24 (7.82%)	
BMI (kg/m^2^)	29.19 ± 8.25	28.80 ± 8.07	31.98 ± 9.00	<.001
≤25 kg/m^2^	948 (38.00%)	868 (39.53%)	80 (27.13%)	
25–30 kg/m^2^	607 (23.62%)	553 (24.35%)	54 (18.42%)	
>30 kg/m^2^	1021 (38.38%)	854 (36.12%)	167 (54.45%)	
WC (m)	0.95 ± 0.19	0.94 ± 0.18	1.03 ± 0.20	<.001
BRI	5.39 ± 2.78	5.26 ± 2.72	6.35 ± 2.99	<.001
Age at menarche (yr)	12.60 ± 1.73	12.62 ± 1.72	12.46 ± 1.81	.26
TG (mmol/l)	1.26 ± 0.79	1.23 ± 0.78	1.45 ± 0.89	<.001
TC (mmol/l)	4.66 ± 0.94	4.65 ± 0.95	4.75 ± 0.89	.14
NHHR	2.33 ± 1.12	2.30 ± 1.12	2.54 ± 1.14	.001
HDL-C (mmol/l)	1.49 ± 0.40	1.50 ± 0.40	1.43 ± 0.40	.02
LDL-C (mmol/l)	2.66 ± 0.80	2.66 ± 0.80	2.69 ± 0.74	.66
Sedentary time(h)	6.37 ± 3.33	6.34 ± 3.31	6.58 ± 3.46	.30
Marital status (%)				<.001
With partner	1676 (67.38%)	1453 (66.02%)	223 (77.13%)	
Without partner	900 (32.62%)	822 (33.98%)	78 (22.87%)	
Sleeping troubles (%)				.00
No	2002 (76.49%)	1791 (77.65%)	211 (68.23%)	
Yes	574 (23.51%)	484 (22.35%)	90 (31.77%)	
Menstrual regularity (%)				.48
Yes	2405 (92.59%)	2120 (92.81%)	285 (90.98%)	
No	171 (7.41%)	155 (7.19%)	16 (9.02%)	
Contraceptive drug use (%)				.42
Yes	1745 (74.80%)	1524 (74.46%)	221 (77.20%)	
No	831 (25.20%)	751 (25.54%)	80 (22.80%)	
Smoking status (%)				.36
Never	1836 (68.06%)	1637 (68.61%)	199 (64.20%)	
Former	285 (12.67%)	242 (12.28%)	43 (15.43%)	
Current	455 (19.27%)	396 (19.12%)	59 (20.37%)	
Diabetes (%)				<.001
No	2479 (96.98%)	2201 (97.55%)	278 (92.92%)	
Yes	97 (3.02%)	74 (2.45%)	23 (7.08%)	
Hypertension (%)				.02
No	2236 (88.19%)	1992 (89.04%)	244 (82.15%)	
Yes	340 (11.81%)	283 (10.96%)	57 (17.85%)	
History of pelvic infection (%)				.01
Yes	122 (4.23%)	99 (3.79%)	25 (7.36%)	
No	2452 (95.77%)	2176 (96.21%)	276 (92.64%)	
Drinking status (%)				.25
No	414 (11.82%)	374 (12.19%)	40 (9.17%)	
Yes	2162 (88.18%)	1901 (87.81%)	261 (90.83%)	
Sedentary behavior (%)				.51
Low sedentary time	1480 (56.63%)	1308 (56.86%)	172 (55.00%)	
High sedentary time	1096 (43.37%)	967 (43.14%)	129 (45.00%)	

The weighted mean ± standard error for continuous variables and unweighted counts (weighted percentage) for categorical variables were calculated.

BMI = body mass index, BRI = body roundness index, HDL-C = high-density lipoprotein cholesterol, LDL-C = low-density lipoprotein cholesterol, NHHR = non - high - density lipoprotein cholesterol/high - density lipoprotein cholesterol, PIR = poverty-to-income ratio, TC = total cholesterol, TG = triglyceride, WC = waist circumference.

* GUID c24d0843-9191-4337-b6f5-d80c87683337

### 3.2. Relationship between BRI and the risk of infertility

Table 2 presents evidence of a positive association between BRI and infertility risk, which was statistically significant across all models. In the crude model, the odds ratio (OR) for infertility per unit increase in BRI was 1.03 (95% CI: 1.07–1.19; *P* = .0001). After adjusting for age and race in Model 1, the OR increased to 1.11 (95% CI: 1.05–1.18; *P* = .0007), and further adjustments in Model 2 yielded an OR of 1.08 (95% CI: 1.02–1.15; *P* = .02). This suggests that with each 1-unit increase in BRI, the risk of infertility rises by 8%. When BRI was divided into tertiles, participants in tertiles 2 (T2) and 3 (T3) exhibited significantly higher infertility risks than those in tertile 1 (T1) (*P* < .05), with a trend indicating an increase in infertility risk with higher BRI tertiles (*P* for trend < .05).

**Table 2:** Association between BRI and female infertility.

	Crude model		Model 1		Model 2	
	OR (95% CI)	*P* value	OR (95% CI)	*P* value	OR (95% CI)	*P* value
BRI	1.13 (1.07–1.19)	<.001	1.11 (1.05–1.18)	<.001	1.08 (1.02–1.15)	.02
T1	Ref.		Ref.		Ref.	
T2	1.91 (1.26–2.90)	.003	1.73 (1.11–2.68)	.02	1.71 (1.03–2.83)	.04
T3	2.98 (1.88–4.70)	<.001	2.68 (1.64–4.39)	<.001	2.46 (1.37–4.43)	.005
*P* for trend		<.001		<.001		.005

Model 1 adjusted for age and race. Model 2 adjusted for age, race, PIR, educational level, marital status and smoking status, hypertension, diabetes, age at menarche, menstrual regularity, TC, HDL-C, drinking status, sleep troubles, sedentary behavior, physical activity, contraceptive drug use, history of pelvic infection.

For the BRI, group T1 is 1.3143 ≤ BRI ≤ 3.8404, group T2 is 3.8406 ≤ BRI ≤ 6.0710, and group T3 is 6.0711 ≤ BRI ≤ 23.4824.

BRI = body roundness index, CI = confidence interval, HDL-C = high-density lipoprotein cholesterol, OR = odds radio, PIR = poverty-to-income ratio, Ref. = reference, TC = total cholesterol.

### 3.3. Weighted RCS and threshold effect analysis

**Figure 2.**
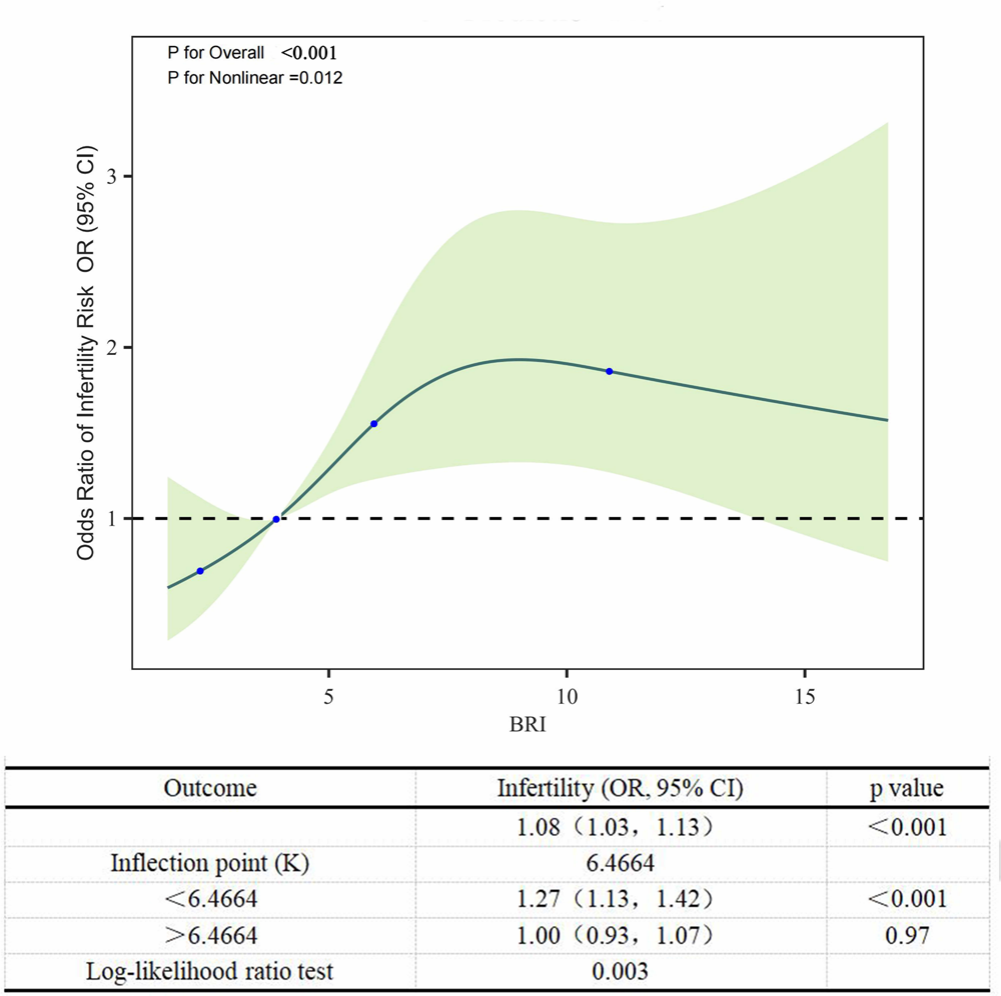
The weighted non-linear association between BRI and female infertility was analyzed using a weighted restricted cubic spline model. Threshold Effect Analysis of BRI and infertility risk using regression analysis of weighted generalized additive model. ORs (blue line) and 95% CI (green shade area) were calculated after adjusted for age, race, PIR, educational level, marital status and smoking status, hypertension, diabetes, age at menarche, menstrual regularity, TC, HDL-C, drinking status, sleep troubles, sedentary behavior, physical activity, contraceptive drug use, history of pelvic infection. The dash line indicated the referent line of OR = 1.

After adjusting for various covariates, the weighted RCS analysis showed a non-linear correlation between BRI and the risk of infertility, with a significant nonlinearity (*P *= .012), as illustrated in Figure 2. Further exploration using weighted generalized additive model regression identified a turning point at a BRI of 6.4664. Below this threshold, the infertility risk notably augmented with increasing BRI (*P* < .001). Nevertheless, when BRI surpassed 6.4664, the correlation between BRI and infertility risk was not statistically significant, as depicted in Figure 2.

### 3.4. Weighted subgroup analysis

**Figure 3.**
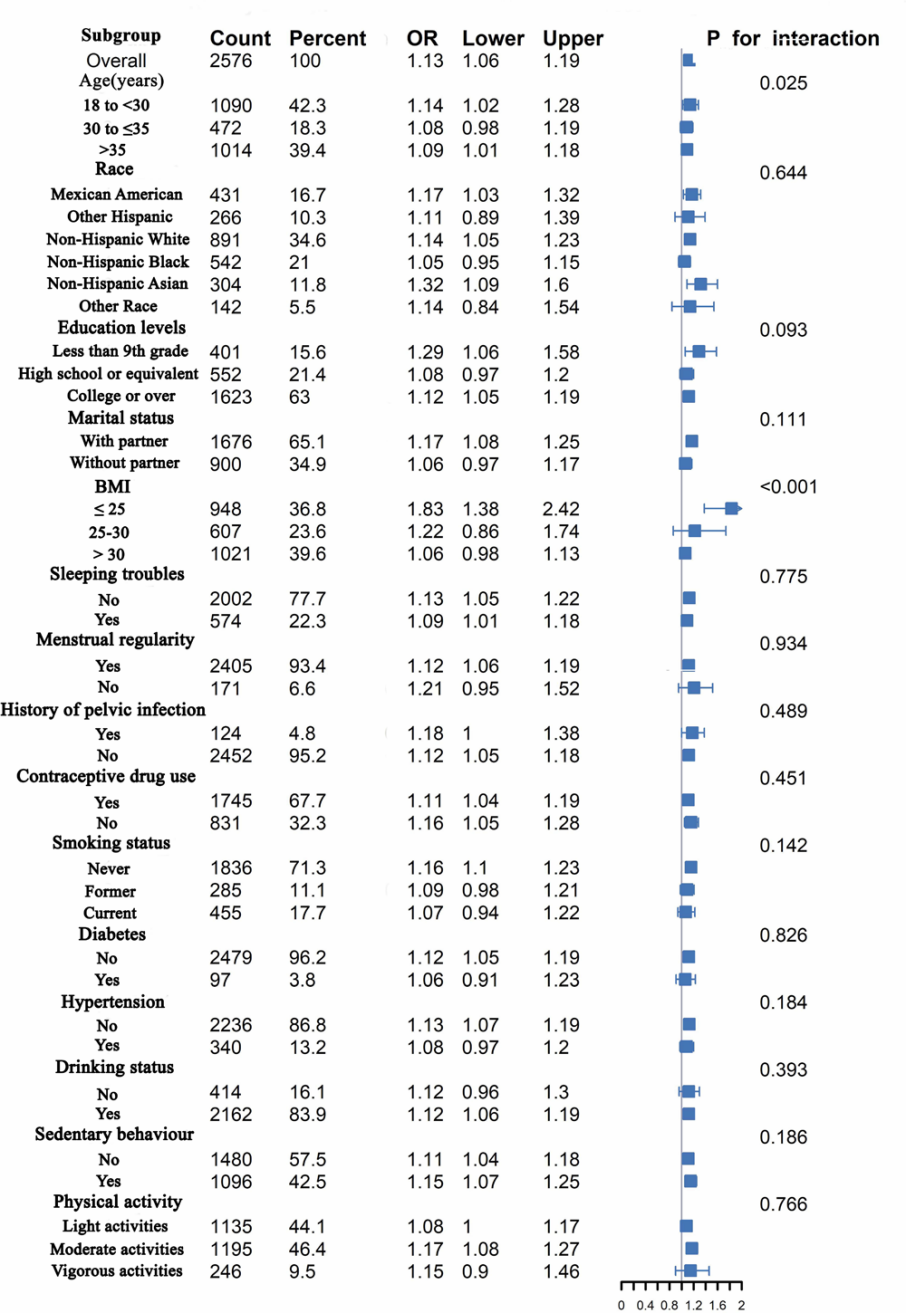
Weighted subgroup and interaction analyses were conducted to examine the associations between BRI and the risk of infertility.

To investigate whether the positive correlation between the BRI and the risk of infertility persists uniformly among diverse subgroups. Across all strata, the risk of infertility demonstrated a trend of increasing with higher BRI (OR > 1). Significant interactions were observed in the age and BMI subgroups, with interaction *P*-values of .025 and < .001, as illustrated in Figure 3. Table S3, Supplemental Digital Content, https://links.lww.com/MD/R17 confirms that the favorable correlation between BRI and the likelihood of infertility remains significant in the 18–30 age group (*P* < .05). Table S4, Supplemental Digital Content, https://links.lww.com/MD/R17 reveals that this positive association is present only in participants with a BMI ≤ 25 kg/m² (*P* = .003).

### 3.5. NHHR as a mediator between BRI and infertility

**Figure 4.**
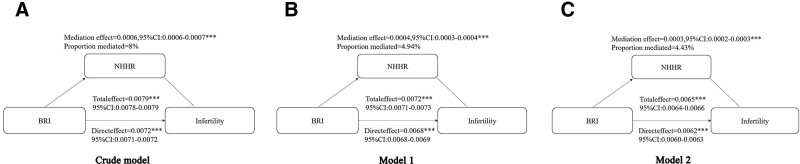
Mediation analysis of the NHHR on the risk of depression and infertility. Model 1 adjusted for age and race. Model 2 adjusted for age, race, PIR, educational level, marital status and smoking status, hypertension, diabetes, age at menarche, menstrual regularity, drinking status, sleep troubles, sedentary behavior, physical activity, contraceptive drug use, history of pelvic infection. ***P < .001.

Table 3 presents the findings from the weighted multivariate linear regression analysis, demonstrating a strong positive correlation between BRI and NHHR after adjusting for all relevant covariates (OR = 1.16; 95% CI: 1.13–1.19; *P *< .001). Table 4 further evaluates the link between NHHR and infertility risk using weighted multivariate logistic regression analysis. NHHR was consistently positively associated with infertility risk across all 3 models, with statistical significance retained in Model 2 (OR = 1.12; 95% CI: 1.00–1.26; *P* = .045). When NHHR was categorized into tertiles for further analysis, infertility risk was notably elevated in T2 and T3 as opposed to tertile T1 across all models (*P *< .05). Trend analysis confirmed a progressive increase in infertility risk with rising NHHR tertiles (*P* for trend < 0.05). Building on these analyses, mediation analysis demonstrated that NHHR partially mediated the connection between BRI and infertility risk. In Model 2, after adjusting for all covariates, NHHR accounted for 4.43% of the linkage between BRI and infertility risk, as illustrated in Figure 4.

**Table 3:** Association between BRI and NHHR.

	Crude model		Model 1		Model 2	
	OR (95% CI)	*P* value	OR (95% CI)	*P* value	OR (95% CI)	*P* value
BRI	1.18 (1.15–1.21)	<.001	1.18 (1.15,1.20)	<.001	1.16 (1.13,1.19)	<.001
T1	Ref.		Ref.		Ref.	
T2	1.99 (1.78–2.24)	<.001	1.94 (1.73,2.18)	<.001	1.86 (1.65,2.09)	<.001
T3	3.24 (2.88–3.66)	<.001	3.20 (2.82,3.65)	<.001	2.90 (2.54,3.31)	<.001
*P* for trend		<.001		<.001		<.001

Model 1 adjusted for age and race. Model 2 adjusted for age, race, PIR, educational level, marital status and smoking status, hypertension, diabetes, age at menarche, menstrual regularity, drinking status, sleep troubles, sedentary behavior, physical activity, contraceptive drug use, history of pelvic infection.

For the BRI, group T1 is 1.3143 ≤ BRI ≤ 3.8404, group T2 is 3.8406 ≤ BRI ≤ 6.0710, and group T3 is 6.0711 ≤ BRI ≤ 23.4824.

BRI = body roundness index, CI = confidence interval, NHHR: = non - high - density lipoprotein cholesterol/high - density lipoprotein cholesterol, OR = odds radio, PIR = poverty-to-income ratio, Ref. = reference.

**Table 4:** Association between NHHR and female infertility.

	Crude model		Model 1		Model 2	
	OR (95% CI)	*P* value	OR (95% CI)	*P* value	OR (95% CI)	*P* value
NHHR	1.18 (1.06–1.32)	.004	1.13 (1.01–1.27)	.03	1.12 (1.00–1.26)	.045
T1	Ref.		Ref.		Ref.	
T2	1.61 (1.07–2.42)	.02	1.61 (1.07–2.42)	.02	1.61 (1.04–2.48)	.03
T3	2.16 (1.46–3.20)	<.001	3.20 (2.82–3.65)	<.001	1.86 (1.22–2.82)	.006
*P* for trend		<.001		.002		.005

Model 1 adjusted for age and race. Model 2 adjusted for age, race, PIR, educational level, marital status and smoking status, age at menarche, menstrual regularity, drinking status, sleep troubles, sedentary behavior, physical activity, contraceptive drug use, history of pelvic infection.

For the NHHR, group T1 is 0.3993 ≤ BRI ≤ 1.7442 group T2 is 1.7448 ≤ BRI ≤ 2.6050, and group T3 is 2.6055 ≤ BRI ≤ 13.9340.

CI = confidence interval, NHHR = non - high - density lipoprotein cholesterol/high - density lipoprotein cholesterol, OR = odds radio, PIR = poverty-to-income ratio, Ref.: = Reference.

### 3.6. Development of a clinical prediction model using machine learning

**Figure 5.**
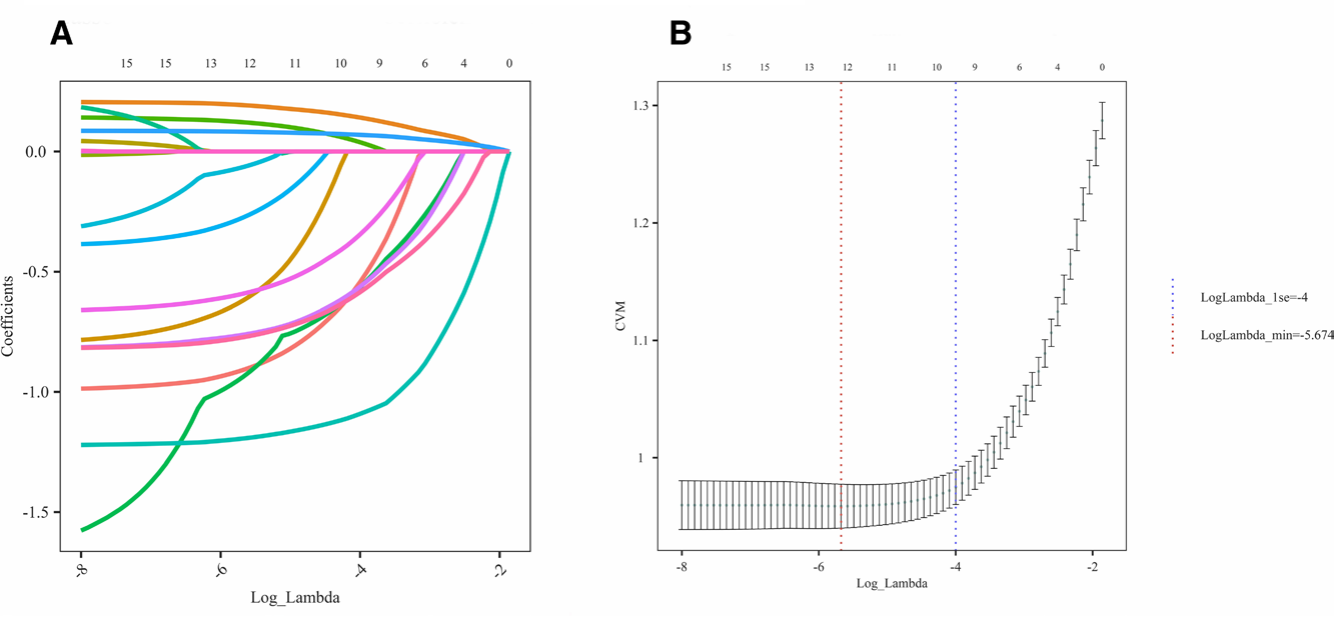
Variable selection using LASSO regression. (A) Regression coefficient profile plot. (B) Cross-validation curve.

**Figure 6.**
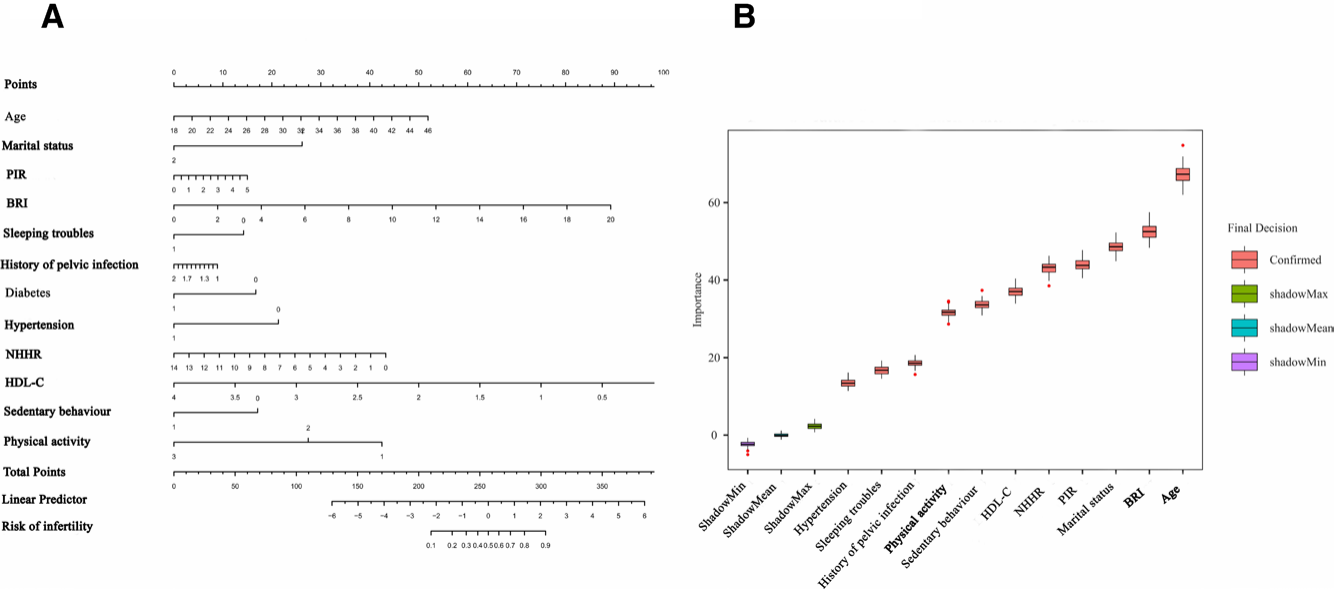
Feature selection. (A) Nomogram of logistic regression. (B) Feature selection using Boruta algorithm.

The dataset was resampled using the synthetic minority oversampling technique method to address class imbalance and was subsequently divided into training (70%) and test (30%) subsets. In the training set, LASSO regression was applied to identify predictive variables, retaining features with non-0 coefficients (Fig. 5). Multivariate logistic regression analysis and the Boruta algorithm, combined with cross-validation(Supplementary Figure 1, Supplemental Digital Content, https://links.lww.com/MD/R17) and clinical relevance, were then used to finalize 6 key predictors: BRI, age, NHHR, PIR, HDL-C, and marital status (Fig. 6). These variables were incorporated into the clinical prediction model.

**Figure 7.**
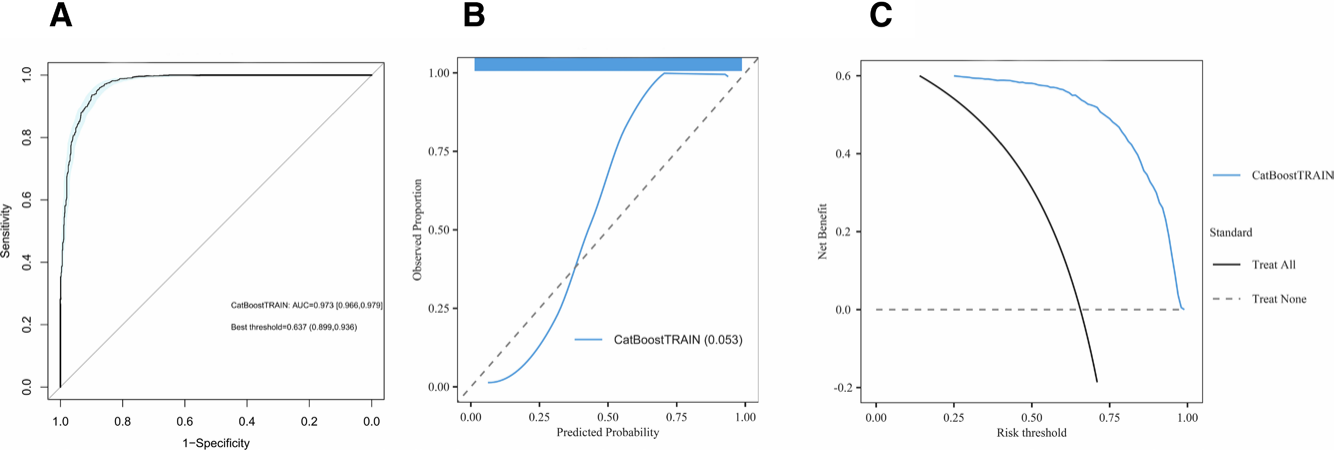
Evaluation of predictive models. A. ROC curve. B. Calibration curve. C. Decision curve analysis (DCA).

The prediction model, built using the CatBoost algorithm, demonstrated excellent discriminative power in the training set, with an area under the ROC curve (AUC) of 0.973 (95% CI: 0.966–0.979) (Fig. 7). Detailed evaluation results are provided in Table S5, Supplemental Digital Content, https://links.lww.com/MD/R17. Figure S2, Supplemental Digital Content, https://links.lww.com/MD/R17 further shows that the model also achieved high predictive performance and clinical risk assessment value in the testing set, confirming its robustness and utility in clinical applications.

## 4. Discussion

This study, based on the NHANES 2013–2018 dataset, employed a cross-sectional design to investigated the relationship between BRI and female infertility risk. The analysis demonstrated a significant positive relationship between BRI and infertility risk, which persisted across various models with or without covariate adjustments. Our analysis demonstrated a significant positive correlation between BRI and NHHR, confirmed by the weighted multivariate regression analysis (OR = 1.16; 95% CI: 1.13–1.19; *P* < .001), suggesting that both variables are closely related. NHHR was identified as a mediator in the relationship between BRI and infertility. Using machine learning techniques, we developed a predictive model incorporating age, BRI, NHHR, PIR, HDL-C, and marital status. This model demonstrated excellent predictive performance in both the training and testing datasets, suggesting its potential utility for clinical infertility risk screening and management.

Previous studies have highlighted the correlation between obesity and various health conditions, including diabetes, hypertension, osteoarthritis, cardiovascular diseases, and gynecological disorders such as endometriosis, ovarian cancer, polycystic ovary syndrome, and menstrual irregularities.^[28–33]^ In the context of assisted reproduction and disease treatment, overweight or obese women often experience poor oocyte quality and unfavorable treatment outcomes. However, weight reduction has been shown to reverse these outcomes, likely due to the restoration of hypothalamic-pituitary-gonadal axis function.^[34–36]^ BMI remains a widely accepted metric for obesity assessment, it has constraints on differentiating fat from muscle mass and in evaluating visceral fat. This has led to the emergence of novel metrics such as BRI, conicity index (C-index), relative fat mass index, which offer superior predictive capability for body fat and visceral fat-related health risks.^[18,37–41]^

BRI, the focus of this study, has had connections with a multitude of health results, including cardiovascular diseases, osteoarthritis, gallstones, nonalcoholic fatty liver disease, and infertility.^[42–46]^ Consistent with findings from Wang et al, our study confirmed a positive association between BRI and infertility risk.^[42]^ Further using weighted RCS analysis, we identified a nonlinear relationship between BRI and infertility risk, with significant associations observed when BRI was below 6.4664 (*P* < .001). Weighted subgroup analyses also revealed interactions by age^[1]^and BMI, with significant associations in individuals aged 18 to 30 years or with a BMI ≤ 25 kg/m² (*P* < .05). NHHR was found to mediate the association between BRI and infertility, suggesting its role as an intermediary factor in this pathway. Incorporating age, NHHR, PIR, and other known risk factors alongside BRI into a predictive model improved the ability to identify high-risk individuals for targeted interventions.

The mechanisms that are at the basis of the relationship between obesity and infertility are complex.

Obese women often experience hypothalamic-pituitary-gonadal axis dysfunction, leading to hormonal imbalances in estradiol, testosterone, luteinizing hormone, and progesterone. These disruptions impair oocyte development, ovulation, and uterine receptivity, contributing to infertility.^[35,36]^ Obesity also induces immune dysregulation and lipid metabolism changes, resulting in steroid hormone synthesis abnormalities that hinder oocyte maturation and disrupt endometrial and follicular development.^[47]^ Abdominal obesity, in particular, predisposes individuals to insulin resistance, hyperinsulinemia, and androgen excess, which further impair follicular development and ovulation.^[47]^ The correlation between BRI and NHHR likely reflects shared mechanisms of lipid metabolism, such as the effects of abdominal obesity, insulin resistance, and dysregulated lipid synthesis, all of which contribute to infertility.

NHHR, as an emerging marker of lipid metabolism, has been extensively studied in diabetes, cardiovascular diseases, and depression. Research by Chen et al found elevated LDL-C and reduced HDL-C levels in overweight or obese women compared to those with normal weight.^[48]^ Song et al reported NHHR as a mediator between central obesity and diabetes.^[49]^ Kerimali Akyildiz and Geng et al demonstrated the positive effects of weight loss interventions, such as increased HDL-C and reduced TC and LDL-C levels, on metabolic health.^[50,51]^ In infertility, cholesterol has been identified as a key factor, with studies showing elevated TC and LDL-C and reduced HDL-C levels in women with infertility compared to those with normal fertility.^[52,53]^

Our study expands on these findings by demonstrating a significant nonlinear association between BRI and infertility risk, particularly when BRI is below 6.4664. Subgroup analysis revealed significant interactions with age and BMI, highlighting distinct risk patterns. NHHR was confirmed as a mediator in the BRI-infertility relationship, and the constructed predictive model provides a valuable tool for clinical risk assessment and management. By incorporating NHHR into the predictive model alongside BRI, age, PIR, and HDL-C, we were able to improve the model’s ability to identify high-risk individuals for infertility, highlighting the value of NHHR in clinical infertility risk screening.

However, this study has limitations. First, several variables were self-reported, potentially introducing recall bias. Second, the NHANES dataset lacks partner fertility information, which may influence infertility outcomes. Lastly, the cros-sectional design prevents causal inferences, necessitating further prospective or cohort studies to confirm these findings.

## 5. Conclusion

BRI is a convenient metric for assessing obesity, and our findings highlight its association with infertility risk, mediated by NHHR. The predictive model developed in this study offers potential clinical utility for infertility risk management and warrants further research for validation and application.

## Acknowledgments

We are grateful to the National Health and Nutrition Examination Survey for the data provided and to all participants for their selfless dedication.

## Author contributions

**Conceptualization:** Weijing Yang, YaLu Fu, XingLong Liu, YuChan Wang, YuHan Meng.

**Data curation:** Weijing Yang, YaLu Fu, XingLong Liu, YuChan Wang, YuHan Meng.

**Formal analysis:** Weijing Yang, YaLu Fu, XingLong Liu, YuChan Wang, YuHan Meng.

**Funding acquisition:** XingLong Liu, YuChan Wang, YuHan Meng.

**Investigation:** Weijing Yang, YaLu Fu, XingLong Liu, YuChan Wang.

**Methodology:** Weijing Yang, XingLong Liu, YuChan Wang.

**Project administration:** Weijing Yang, YaLu Fu, XingLong Liu, YuChan Wang, YuHan Meng.

**Resources:** Weijing Yang, XingLong Liu, YuChan Wang.

**Software:** Weijing Yang, YaLu Fu, XingLong Liu, YuChan Wang.

**Supervision:** Weijing Yang, YaLu Fu, XingLong Liu, YuChan Wang, YuHan Meng.

**Validation:** Weijing Yang, XingLong Liu, YuChan Wang, YuHan Meng.

**Visualization:** Weijing Yang, XingLong Liu, YuChan Wang.

**Writing – original draft:** Weijing Yang, YaLu Fu, XingLong Liu, YuChan Wang.

**Writing – review & editing:** Weijing Yang, XingLong Liu, YuChan Wang, XiangYan Li, ZhanHong Du, YuHan Meng.

## Supplementary Material


