# Supplementary material for: Nonlinear association of body roundness index with female infertility and the mediating effect of NHHR: A cross-sectional study
Source: Medicine (Baltimore). 2025 Dec 19;104(51):e46768. doi: 10.1097/MD.0000000000046768 (PMC12727249; doi:10.1097/MD.0000000000046768)

| **Supplementary Table 1** Characteristics of selected participants from the NHANES 2013–2018 (weighted analysis). | | | | |
| --- | --- | --- | --- | --- |
| Characteristic | Overall N = 38981556 | Fertile  N = 34180892 | Infertile  N = 4800664 | *P*-value |
| Age(years) | 31.59 ± 7.71 | 31.11 ± 7.70 | 35.00 ± 6.94 | <0.001 |
| 18 to ＜30 | 16,980,420 (43.56%) | 15,847,980 (46.37%) | 1,132,439 (23.59%) |  |
| 30 to ≤35 | 7,022,431 (18.01%) | 6,035,178 (17.66%) | 987,254 (20.56%) |  |
| ＞35 | 14,978,705 (38.43%) | 12,297,734 (35.98%) | 2,680,971 (55.85%) |  |
| Race(%) |  |  |  | 0.600 |
| Mexican American | 4,441,294 (11.39%) | 3,958,535 (11.58%) | 482,760 (10.06%) |  |
| Other Hispanic | 2,943,216 (7.55%) | 2,649,232 (7.75%) | 293,983 (6.12%) |  |
| Non-Hispanic White | 22,473,989 (57.65%) | 19,490,368 (57.02%) | 2,983,621 (62.15%) |  |
| Non-Hispanic Black | 4,955,816 (12.71%) | 4,360,489 (12.76%) | 595,326 (12.40%) |  |
| Non-Hispanic Asian | 2,249,082 (5.77%) | 2,019,508 (5.91%) | 229,575 (4.78%) |  |
| Other Race | 1,918,159 (4.92%) | 1,702,760 (4.98%) | 215,399 (4.49%) |  |
| Education levels (%) |  |  |  | 0.92 |
| Less than 9th grade | 4,407,188 (11.31%) | 3,894,248 (11.39%) | 512,940 (10.68%) |  |
| High school or equivalent | 7,852,023 (20.14%) | 6,895,379 (20.17%) | 956,644 (19.93%) |  |
| College or over | 26,722,345 (68.55%) | 23,391,266 (68.43%) | 3,331,080 (69.39%) |  |
| PIR  Physical activity | 2.69 ± 1.65 | 2.66 ± 1.65 | 2.89 ± 1.66 | 0.05 |
|  |  |  |  | 0.340 |
| **Light activities** | 15,098,193 (38.73%) | 13,017,936 (38.09%) | 2,080,257 (43.33%) |  |
| Moderate activities | 20,066,087 (51.48%) | 17,720,991 (51.84%) | 2,345,095 (48.85%) |  |
| Vigorous activities | 3,817,276 (9.79%) | 3,441,965 (10.07%) | 375,312 (7.82%) |  |
| BMI( kg/m^2^) | 29.19 ± 8.25 | 28.80 ± 8.07 | 31.98 ± 9.00 | <0.001 |
| ≤ 25 kg/m^2^ | 14,814,798 (38.00%) | 13,512,302 (39.53%) | 1,302,496 (27.13%) |  |
| 25-30 kg/m^2^ | 9,206,554 (23.62%) | 8,322,341 (24.35%) | 884,214 (18.42%) |  |
| > 30 kg/m^2^ | 14,960,204 (38.38%) | 12,346,250 (36.12%) | 2,613,954 (54.45%) |  |
| WC(m) | 0.95 ± 0.19 | 0.94 ± 0.18 | 1.03 ± 0.20 | <0.001 |
| BRI | 5.39 ± 2.78 | 5.26 ± 2.72 | 6.35 ± 2.99 | <0.001 |
| Age at menarche (years) | 12.60 ± 1.73 | 12.62 ± 1.72 | 12.46 ± 1.81 | 0.26 |
| TG(mmol/l) | 1.26 ± 0.79 | 1.23 ± 0.78 | 1.45 ± 0.89 | <0.001 |
| TC(mmol/l) | 4.66 ± 0.94 | 4.65 ± 0.95 | 4.75 ± 0.89 | 0.14 |
| NHHR | 2.33 ± 1.12 | 2.30 ± 1.12 | 2.54 ± 1.14 | 0.00 |
| HDL-C(mmol/l) | 1.49 ± 0.40 | 1.50 ± 0.40 | 1.43 ± 0.40 | 0.02 |
| LDL-C(mmol/l) | 2.66 ± 0.80 | 2.66 ± 0.80 | 2.69 ± 0.74 | 0.66 |
| Sedentary time（hours） | 6.37 ± 3.33 | 6.34 ± 3.31 | 6.58 ± 3.46 | 0.30 |
| Marital status (%) |  |  |  | <0.001 |
| With partner | 26,267,564 (67.38%) | 22,564,678 (66.02%) | 3,702,886 (77.13%) |  |
| Without partner | 12,713,992 (32.62%) | 11,616,214 (33.98%) | 1,097,778 (22.87%) |  |
| Sleeping troubles |  |  |  | 0.00 |
| No | 29,818,270 (76.49%) | 26,542,583 (77.65%) | 3,275,687 (68.23%) |  |
| Yes | 9,163,285 (23.51%) | 7,638,309 (22.35%) | 1,524,976 (31.77%) |  |
| Menstrual regularity (%) |  |  |  | 0.48 |
| Yes | 36,091,511 (92.59%) | 31,723,731 (92.81%) | 4,367,781 (90.98%) |  |
| No | 2,890,044 (7.41%) | 2,457,161 (7.19%) | 432,883 (9.02%) |  |
| Contraceptive drug use |  |  |  | 0.42 |
| Yes | 29,156,473 (74.80%) | 25,450,373 (74.46%) | 3,706,101 (77.20%) |  |
| No | 9,825,083 (25.20%) | 8,730,520 (25.54%) | 1,094,563 (22.80%) |  |
| Smoking status (%) |  |  |  | 0.36 |
| Never | 26,531,820 (68.06%) | 23,449,914 (68.61%) | 3,081,906 (64.20%) |  |
| Former | 4,937,620 (12.67%) | 4,196,761 (12.28%) | 740,859 (15.43%) |  |
| Current | 7,512,115 (19.27%) | 6,534,217 (19.12%) | 977,899 (20.37%) |  |
| Diabetes (%) |  |  |  | <0.001 |
| No | 37,805,699 (96.98%) | 33,344,740 (97.55%) | 4,460,959 (92.92%) |  |
| Yes | 1,175,856 (3.02%) | 836,152 (2.45%) | 339,704 (7.08%) |  |
| Hypertension (%) |  |  |  | 0.02 |
| No | 34,378,877 (88.19%) | 30,435,190 (89.04%) | 3,943,686 (82.15%) |  |
| Yes | 4,602,679 (11.81%) | 3,745,702 (10.96%) | 856,977 (17.85%) |  |
| History of pelvic infection |  |  |  | 0.01 |
| Yes | 1,650,086 (4.23%) | 1,296,830 (3.79%) | 353,256 (7.36%) |  |
| No | 37,331,469 (95.77%) | 32,884,062 (96.21%) | 4,447,408 (92.64%) |  |
| Drinking status |  |  |  | 0.25 |
| No | 4,606,223 (11.82%) | 4,166,093 (12.19%) | 440,130 (9.17%) |  |
| Yes | 34,375,333 (88.18%) | 30,014,799 (87.81%) | 4,360,534 (90.83%) |  |
| Sedentary behaviour |  |  |  | 0.51 |
| Low sedentary time | 22,073,935 (56.63%) | 19,433,604 (56.86%) | 2,640,330 (55.00%) |  |
| High sedentary time | 16,907,621 (43.37%) | 14,747,288 (43.14%) | 2,160,334 (45.00%) |  |
|  | | | | |

#GUID c24d0843-9191-4337-b6f5-d80c87683337

| **Supplementary Table 2** Unweighted characteristics of selected participants from the NHANES 2013–2018. | | | | | | |
| --- | --- | --- | --- | --- | --- | --- |
| Characteristic | level | Overall | Missing | Fertile | Infertile | p |
| n |  | 2576 |  | 2275 | 301 |  |
| Age(years) |  | 31.69±7.89 | 0.0 | 31.28±7.91 | 34.75±6.96 | <0.01 |
| Age (%) | 18 to ＜30 | 1090 (42.31) | 0.0 | 1013 (44.53) | 77 (25.58) | <0.01 |
|  | 30 to ≤35 | 472 (18.32) |  | 415 (18.24) | 57 (18.94) |  |
|  | ＞35 | 1014 (39.36) |  | 847 (37.23) | 167 (55.48) |  |
| Race (%) | Mexican American | 431 (16.73) | 0.0 | 384 (16.88) | 47 (15.61) | 0.58 |
|  | Other Hispanic | 266 (10.33) |  | 242 (10.64) | 24 (7.97) |  |
|  | Non-Hispanic White | 891 (34.59) |  | 775 (34.07) | 116 (38.54) |  |
|  | Non-Hispanic Black | 542 (21.04) |  | 478 (21.01) | 64 (21.26) |  |
|  | Non-Hispanic Asian | 304 (11.80) |  | 269 (11.82) | 35 (11.63) |  |
|  | Other Race | 142 (5.51) |  | 127 (5.58) | 15 (4.98) |  |
| Education levels(%) | Less than 9th grade | 401 (15.57) | 0.0 | 360 (15.82) | 41 (13.62) | 0.29 |
|  | High school or equivalent | 552 (21.43) |  | 494 (21.71) | 58 (19.27) |  |
|  | College or over | 1623 (63.00) |  | 1421 (62.46) | 202 (67.11) |  |
| PIR |  | 2.33±1.6 | 0.0 | 2.29±1.59 | 2.58±1.66 | <0.01 |
| Physical activity (%) | **Light activities** | 1135 (44.06) | 0.0 | 990 (43.52) | 145 (48.17) | 0.26 |
|  | Moderate activities | 1195 (46.39) |  | 1063 (46.73) | 132 (43.85) |  |
|  | Vigorous activities | 246 (9.55) |  | 222 (9.76) | 24 (7.97) |  |
| BMI( kg/m^2^) |  | 29.29±8.24 | 0.0 | 28.93±8.08 | 31.98±8.96 | <0.01 |
| BMI (%) | ≤ 25 kg/m^2^ | 948 (36.80) | 0.0 | 868 (38.15) | 80 (26.58) | <0.01 |
|  | 25-30 kg/m^2^ | 607 (23.56) |  | 553 (24.31) | 54 (17.94) |  |
|  | > 30 kg/m^2^ | 1021 (39.64) |  | 854 (37.54) | 167 (55.48) |  |
| WC(m) |  | 0.95±0.19 | 0.0 | 0.94±0.18 | 1.02±0.2 | <0.01 |
| BRI |  | 5.46±2.77 | 0.0 | 5.34±2.72 | 6.37±2.98 | <0.01 |
| Age at menarche (years) |  | 12.55±1.78 | 0.0 | 12.57±1.76 | 12.41±1.94 | 0.14 |
| TG(mmol/l) |  | 1.27±0.84 | 36.9 | 1.25±0.83 | 1.42±0.92 | 0.01 |
| TC(mmol/l) |  | 4.64±0.94 | 0.0 | 4.63±0.94 | 4.74±0.91 | 0.06 |
| NHHR |  | 2.38±1.18 | 0.0 | 2.35±1.17 | 2.62±1.18 | <0.01 |
| HDL-C(mmol/l) |  | 1.47±0.4 | 0.0 | 1.48±0.4 | 1.4±0.38 | <0.01 |
| LDL-C(mmol/l) |  | 2.66±0.70 | 55.2 | 2.65±0.79 | 2.73±0.77 | 0.43 |
| Sedentary time（hours） |  | 6.24±3.38 | 0.0 | 6.22±3.37 | 6.35±3.51 | 0.53 |
| Marital status (%) | With partner | 1676 (65.06) | 0.0 | 1453 (63.87) | 223 (74.09) | <0.01 |
|  | Without partner | 900 (34.94) |  | 822 (36.13) | 78 (25.91) |  |
| Sleeping troubles(%) | No | 2002 (77.72) | 0.0 | 1791 (78.73) | 211 (70.10) | <0.01 |
|  | Yes | 574 (22.28) |  | 484 (21.27) | 90 (29.90) |  |
| Menstrual regularity (%) | Yes | 2405 (93.36) | 0.0 | 2120 (93.19) | 285 (94.68) | 0.39 |
|  | No | 171 (6.64) |  | 155 (6.81) | 16 (5.32) |  |
| Contraceptive drug use(%) | Yes | 1745 (67.74) | 0.0 | 1524 (66.99) | 221 (73.42) | 0.03 |
|  | No | 831 (32.26) |  | 751 (33.01) | 80 (26.58) |  |
| Smoking status (%) | Never | 1836 (71.27) | 0.0 | 1637 (71.96) | 199 (66.11) | 0.07 |
|  | Former | 285 (11.06) |  | 242 (10.64) | 43 (14.29) |  |
|  | Current | 455 (17.66) |  | 396 (17.41) | 59 (19.60) |  |
| Diabetes (%) | No | 2479 (96.23) | 0.0 | 2201 (96.75) | 278 (92.36) | <0.01 |
|  | Yes | 97 (3.77) |  | 74 (3.25) | 23 (7.64) |  |
| Hypertension (%) | Yes | 340 (13.20) | 0.0 | 283 (12.44) | 57 (18.94) | <0.01 |
|  | No | 2236 (86.80) |  | 1992 (87.56) | 244 (81.06) |  |
| History of pelvic infection(%) | Yes | 124 (4.81) | 0.0 | 99 (4.35) | 25 (8.31) | <0.01 |
|  | No | 2452 (95.19) |  | 2176 (95.65) | 276 (91.69) |  |
| Drinking status(%) | No | 414 (16.07) | 0.0 | 374 (16.44) | 40 (13.29) | 0.19 |
|  | Yes | 2162 (83.93) |  | 1901 (83.56) | 261 (86.71) |  |
| Sedentary behaviour(%) | Low sedentary time | 1480 (57.45) | 0.0 | 1308 (57.49) | 172 (57.14) | 0.96 |
|  | High sedentary time | 1096 (42.55) |  | 967 (42.51) | 129 (42.86) |  |
|  | | | | | | |

**Supplementary Table 3** Association between BRI and female infertility stratified by age.

| **Age（years）** | **Crude model** | | **Model 1** | | **Model 2** | |
| --- | --- | --- | --- | --- | --- | --- |
|  | **OR (95% CI)** | ***P* value** | **OR (95% CI)** | ***P* value** | **OR (95% CI)** | ***P* value** |
| 18 to ＜30 | 1.20(1.10,1.32) | <0.001 | 1.19(1.08,1.32) | <0.001 | 1.14(1.00,1.30) | 0.04 |
| 30 to ≤35 | 1.10(1.01,1.20) | 0.04 | 1.10(0.996,1.22) | 0.06 | 1.04(0.92,1.18) | 0.52 |
| ＞35 | 1.07(0.99,1.15) | 0.08 | 1.08(0.999,1.16) | 0.05 | 1.07(0.97,1.17) | 0.15 |

Model 1 adjusted for race.

Model 2 adjusted for race, PIR, educational level, marital status and smoking status, hypertension, diabetes, age at menarche, menstrual regularity, TC, HDL-C, drinking status, sleep troubles, sedentary behaviour, physical activity, contraceptive drug use, history of pelvic infection.

OR: Odds radio, CI: Confidence interval, Ref.: Reference, PIR:poverty-to-income ratio, BRI:body roundness index,TC:total cholesterol, HDL-C:high-density lipoprotein cholesterol.

**Supplementary Table 4** Association between BRI and female infertility stratified by BMI.

| **BMI（kg/m^2^）** | **Crude model** | | **Model 1** | | **Model 2** | |
| --- | --- | --- | --- | --- | --- | --- |
|  | **OR (95% CI)** | ***P* value** | **OR (95% CI)** | ***P* value** | **OR (95% CI)** | ***P* value** |
| **≤25** | 1.93(1.39,2.69) | <0.001 | 2.05(1.45,2.91) | ＜0.001 | 1.75(1.24,2.46) | 0.003 |
| **25 to ＜30** | 1.16(0.82,1.65) | 0.40 | 1.16(0.82,1.64) | 0.38 | 1.15(0.77,1.72) | 0.49 |
| **≥30** | 1.04(0.97,1.13) | 0.24 | 1.04(0.97,1.12) | 0.28 | 1.01(0.93,1.10) | 0.77 |

Model 1 adjusted for race.

Model 2 adjusted for race, PIR, educational level, marital status and smoking status, hypertension, diabetes, age at menarche, menstrual regularity, TC, HDL-C, drinking status, sleep troubles, sedentary behaviour, physical activity, contraceptive drug use, history of pelvic infection.

OR: Odds radio, CI: Confidence interval, Ref.: Reference, PIR:poverty-to-income ratio, BRI:body roundness index,TC:total cholesterol, HDL-C:high-density lipoprotein cholesterol.

| **Supplementary Table 5** Performance Metrics of the Prediction Model Built with the CatBoost Algorithm, Including AUC, Accuracy, Recall, and F1-Score. | | | | | | | | | |
| --- | --- | --- | --- | --- | --- | --- | --- | --- | --- |
| Accuracy | Prevalence | Recall | F1-Score | MCC | AUROC | Presicion | Specificity | FNR | FPR |
| 0.925 | 0.656 | 0.989 | 0.945 | 0.834 | 0.973 | 0.905 | 0.803 | 0.011 | 0.197 |
|  | | | | | | | | | |

**Supplementary Figure 1** Evaluation of predictive models based on cross-validation results: A. ROC curve. B. Calibration curve. C. Decision curve analysis (DCA).


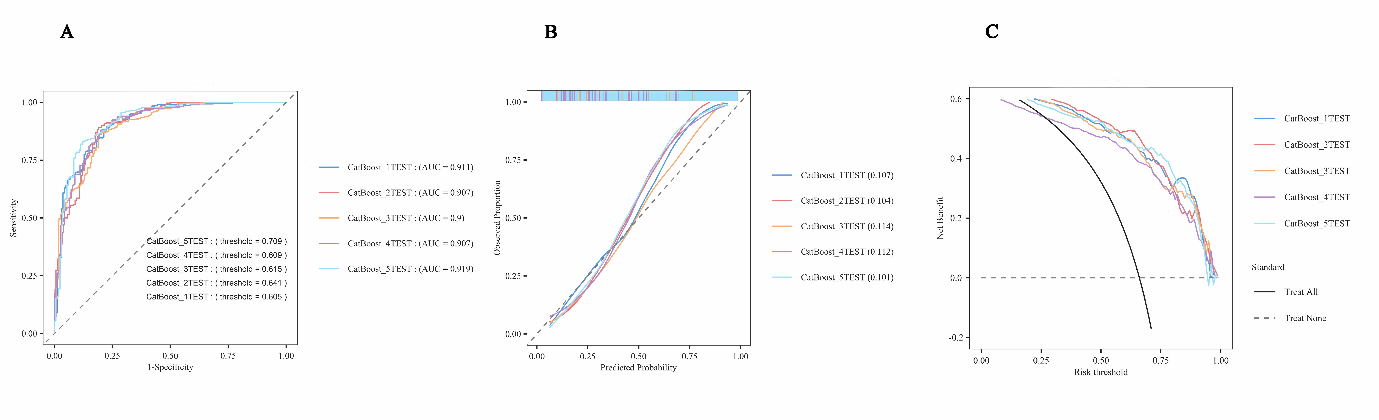


**Supplementary Figure 2** Evaluation of predictive models on the test set: A. ROC curve. B. Calibration curve. C. Decision curve analysis (DCA) on the test set.


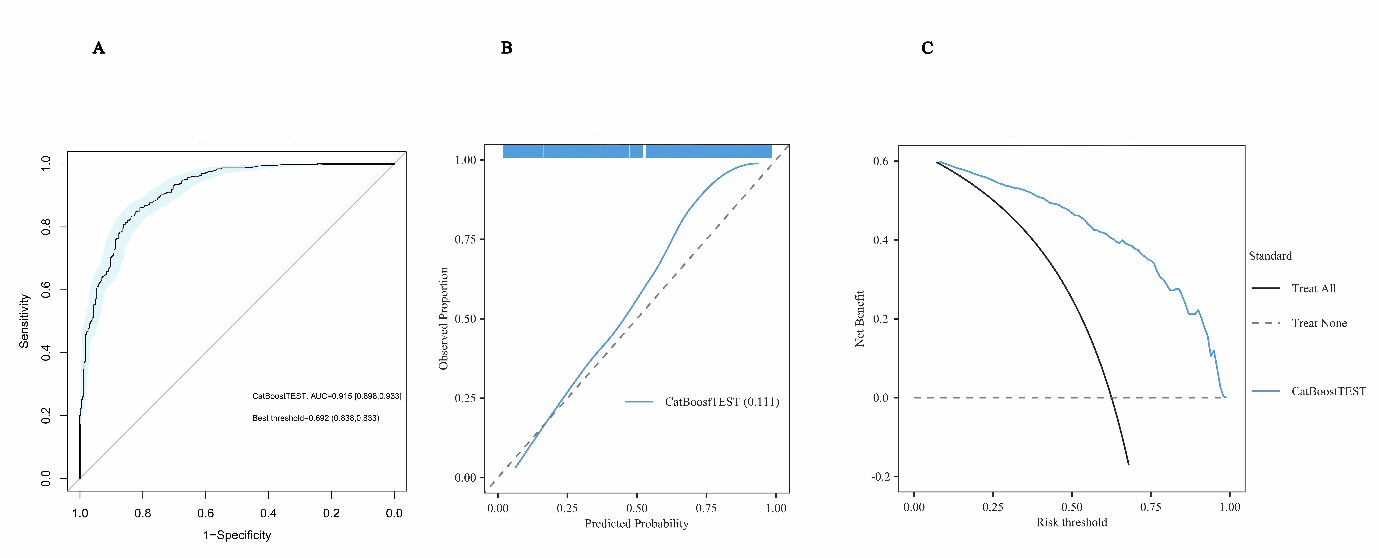

Supplement: Supplementary file 1 [file medi-104-e46768-s001.docx]
